# Successful pandemic management through computer science: a case study of a financial corporation with workers on premises

**DOI:** 10.3389/fpubh.2023.1208751

**Published:** 2023-11-17

**Authors:** Angélica Partida-Hanon, Ramón Díaz-Garrido, José María Mendiguren-Santiago, Laura Gómez-Paredes, Juan Muñoz-Gutiérrrez, María Antonia Miguel-Rodríguez, Luis Reinoso-Barbero

**Affiliations:** ^1^Department of Advanced Analytics, Santander Corporate and Investment Banking, Madrid, Spain; ^2^Department of Quantitative Methods, Universidad Loyola Andalucía, Córdoba, Spain; ^3^Department of Health and Occupational Risk Prevention, Banco Santander, Madrid, Spain; ^4^Faculty of Health Sciences, International University of La Rioja, La Rioja, Spain

**Keywords:** health informatics, occupational and industrial medicine, epidemiology, information management, COVID-19

## Abstract

**Background:**

In November 2019, an infectious agent that caused a severe acute respiratory illness was first detected in China. Its rapid spread resulted in a global lockdown with negative economic impacts. In this regard, we expose the solutions proposed by a multinational financial institution that maintained their workers on premises, so this methodology can be applied to possible future health crisis.

**Objectives:**

To ensure a secure workplace for the personnel on premises employing biomedical prevention measures and computational tools.

**Methods:**

Professionals were subjected to recurrent COVID-19 diagnostic tests during the pandemic. The sanitary team implemented an individual following to all personnel and introduced the information in databases. The data collected were used for clustering algorithms, decision trees, and networking diagrams to predict outbreaks in the workplace. Individualized control panels assisted the decision-making process to increase, maintain, or relax restrictive measures.

**Results:**

55,789 diagnostic tests were performed. A positive correlation was observed between the cumulative incidence reported by Madrid’s Ministry of Health and the headcount. No correlation was observed for occupational infections, representing 1.9% of the total positives. An overall 1.7% of the cases continued testing positive for COVID-19 after 14 days of quarantine.

**Conclusion:**

Based on a combined approach of medical and computational science tools, we propose a management model that can be extended to other industries that can be applied to possible future health crises. This work shows that this model resulted in a safe workplace with a low probability of infection among workers during the pandemic.

## Introduction

### Background

On January 30, 2020, the World Health Organization declared the 2019-SARS-CoV-2 outbreak in China as a Public Health Emergency of International Importance (1). Several national territories imposed strict confinement on their population to limit the transmission of the infectious agent, resulting in half of the world’s population being on lockdown by April 2020 (2, 3).

This abrupt shift toward a fulltime teleworking strategy resulted in several negative impacts on mental and physical health, productivity, and workforce engagement caused by reduced social interactions and physical activity (4–7). Additionally, the confinement resulted in a demand shock that evolved into a worldwide economic crisis (8, 9).

### Context of this study

At the beginning of the lockdown in Spain, financial companies and other professional sectors had to adhere to the protocols defined by the Spanish Ministry of Health (10). Due to the challenge to comply with the norm and continuing the business as usual, professional activities that were not considered as essential (11), ceased their activities or decreed teleworking to their personnel. In this regard, the financial sector was considered essential by the government, and during the lockdown, employees had special permission to move to the workplace (11).

Banco Santander is a leading commercial bank founded in 1857 and headquartered in Spain. It has a meaningful presence in 10 core markets in Europe, North America, and South America, and is one of the largest banks in the world by market capitalization (12, 13). Due to the business’s characteristics, the entity’s international projection, and employees’ frequent business trips, the corporate center’s population is susceptible to pathogenic organisms endemic to other geographies (14). The airborne transmission of infectious agents is also significant in an office-based environment, where several people come together in the same closed space for a prolonged period (14).

Before, during, and after the health crisis caused by SARS-CoV-2, multiple management committees were organized at the entity’s Headquarters (HQ), and diverse protocols were adjusted according to the evolution of the pandemic in the region to guarantee a safe workplace and provide continuity to the business. Therefore, to minimize the adverse effects on health and the economy, the entity invested several resources to maintain an equilibrium between its economic activity and occupational safety.

This work is focused on the description of the combined measures adopted in the corporate center (HQ) of Banco Santander located in Spain, by including the basic physical prevention strategies (facemasks, screens, social distancing, and testing), followed by an analysis of the evolution of the pandemic within the studied population (seasonal effects, comparison with the overall region incidence), and finishing with workplace diagnosis assisted by data science: detection of infection hotspots through networking diagrams.

It is worth highlighting that all preventive measures were continuously adapted based on the evolution of the pandemic, the availability of diagnostic tests, and the regional limitations imposed by the government.

### Basic prevention measures

Banco Santander HQ is located at the offices of Ciudad Grupo Santander in the municipality of Boadilla del Monte, Community of Madrid, Spain. By June 2022, HQ had an internal headcount of 11,322 employees, being 4,561 (40.3%) women and 6,761 (59.7%) men.

On January 31, 2020, the first confirmed case of COVID-19 in Spain was diagnosed (15), while on February 13, 2020, the first death from COVID-19 in Spain occurred (16). On March 14, 2020, the government declared the first State of Alarm throughout the national territory due to the spread of the disease.

Given the rapid expansion of the viral agent, an announcement from Human Resources (HR) was sent to all employees on March 3, 2020. HR advisors included the replacement of unnecessary travel and meetings with videoconferences, avoiding going to the workplace if symptoms compatible with the disease were present, and restricting visits from personnel outside the corporate center. Subsequently, prior to the declaration of international pandemic by the World Health Organization (1) and the declaration of the state of alarm by the Government of Spain (2), remote working was mandatory for less critical workers.

Between April 2020 and March 2022, facemasks and social distancing were mandatory. The company facilitated masks, screens, and sanitizing gel kits, and increased the frequency of cleaning and air renovation. Additionally, the sanitary team broadcasted 13 webinars to update the evolution of the pandemic, how their data are being used and answer the most frequent questions from employees.

Three different communication lines were available for employees to inform the medical staff of relevant changes in their health status due to: (1) the presence of COVID-compatible symptoms, (2) a recent contact with a COVID-positive person, (3) being COVID positive, and (4) having health risk-associated factors.

Three vaccination campaigns were organized: two flu campaigns during the fall and winter of 2020 and 2021, and one against COVID-19 during the summer of 2021. Employees were the target population for the flu vaccinations, while the COVID-19 campaign focused on the general population and employees.

## Methods and computational tools

Three main databases were created in April, 2020, with the following information: (1) tests and results, (2) medical checkups, and (3) contact-tracking relationships. The fields were updated depending on the evolution of the pandemic with new information such as COVID-19 variants (from October, 2020), antigen tests (from August, 2020), trips, and occupational contacts (from July, 2020).

Due to the availability of diagnostic tests for current infections in the general population starting in April 2020, infections were not included in this study prior to this date. Infections after April 2022, were also excluded because of the relaxation of the measures and lack of data.

Three main coordination teams were created to register and manage the health-related information: (1) An internal medical team composed by four doctors and four nurses who were legally authorized to take biological extractions, tests and fill the health-related information through patient interview and examinations, as well, to keep tracking of the medical cases. (2) A contact tracing team (tracking team) composed by five healthcare and HR professionals who were legally authorized to identify the infected patients and notify the personnel if they were in contact with a COVID-positive person. The team also reported the number of infections and COVID-19 contacts to the Spanish Ministry of Health. (3) A technological team composed by an expert that created and maintained the databases, reporting dashboards, programmed the algorithms to keep an update of the general health status and actioned alarms if there were local outbreaks detected in the buildings. It is worth highlighting that patient confidentiality was strictly maintained at all levels during the whole process, including the contact-tracing notifications and the information reported was grouped and anonymized.

When employees informed the staff about changes in their health status, a medical professional evaluated each case individually and filled a database with the following relevant health information: consultation date, symptoms, test results, date of contact with an infected patient, health-risk associated factors, list of contacts, date of infection, hospitalization, required intensive care unit (ICU), contact type (occupational or non-occupational), travel, and death. Access to the offices was restricted for individuals with confirmed or high risk of infection: simultaneously, the medical staff informed the employees if they had been in contact with a COVID-positive person. The definition of a COVID-19 contact was established and continuously revised by the Spanish Ministry of Health, depending on the virus strain and scientific research (10).

Once the first infection was recorded in July 2020, the medical team performed a manual contact-tracing search through patient interviews by personally asking if they can identify the colleagues who shared space with them during a specific time range. Additionally, an algorithm suggested a list of workers who shared the same space, manager, or team, and could coincide with a positive worker within a time range.

The medical and tracking staff later analyzed the suggested list to double check the possibility of coincidence; if confirmed, the contact was quarantined and added to the list of confirmed contacts. This suggestion was manually removed in the case of zero possibility of contact (e.g., 100% teleworking in the time range).

The medical and tracking staff filled a table with all the identified close contacts; if the contact was not due to occupational reasons, such as sharing the same residence, it was considered non-occupational.

Occupational infections were considered in the following cases: (1) If the COVID-positive worker that got infected had access to the office within a defined period before the symptoms or diagnosis and had close contact with another previously identified positive worker. (2) In the previous case, but with no precisely identified contact from personal life, and if another COVID-positive worker shared space during the time range. The period was adjusted based on the known incubation time of the virus and strain. By default, the time range was defined as 21 days before the appearance of the first symptoms (if known) or the first positive test result. Two types of networking diagrams were programmed: one derived from the list suggested by the algorithm and another derived from the list with the confirmed contacts to illustrate the contact lists and detect a possible infection hotspot. Nodes and their relationships by links represent workers. The Ministry of Health has defined an occupational outbreak as a grouping of three or more cases of active infection with an epidemiological link (10). Outbreaks were visually detected from the networking diagram of the confirmed contacts.

Thirty-seven control panels with real-time information were programmed in R markdown and published as HTML files, which allowed different professionals to interact with the data and filter the information. Different levels of confidentiality were applied: medical staff had full access to personal records and lists to make proper individual followings, while the board only had access to cumulated and anonymized data.

Daily emails were sent to the tracking and medical team with relevant information on the development of the virus: number of contacts, workers on premises, number of infections, quarantines, infection hotspots, graphs, and pending following.

The programming language R 3.6.2 was the main language used for statistical analyses (17).

The magnitude of the positive cases was interpreted based on the cumulative incidence (CI) during the last 7 or 14 days, and calculated as follows: the number of newly diagnosed COVID-positive infections during the last 14 days, divided by the number of people free of the infection at the beginning of the period; then, multiplied by 100,000.

Student’s *t*-test and Fisher’s F-test were used to evaluate the differences of means and variances between the Incidences in the Community of Madrid and Headquarters as a function of time. Pearson’s correlation test was used to analyze the correlations between the series.

The limits of statistical differences were determined with *p* values below 0.05, with a confidence interval of 95%.

### Return strategies

The strictest confinement ended on April 27, 2020, to reach the “new normality.” Softer confinement measures were then imposed, ending on June 22 of the same year.

Once the strictest confinement ended, the less critical staff members gradually returned to the premises. Preselection was carried out by prioritizing people who did not have associated health risk factors, presented teleworking limitations, and could reconcile personal and familial responsibilities. Prior to the return and to minimize the possibility of incorporating employees with current infections, the entire headcount had to pass a medical examination that included available and validated diagnostic tests, such as Real-Time Quantitative Reverse Transcription PCR (qRT-PCR) or antigen (when available) tests. This protocol has also been applied to new staff additions.

Three massive test screenings were performed depending on critical moments of high personal flow, such as return from holidays: (1) qRT-PCR and antibody tests during the post-confinement return between April 3 and August 24, 2020. (2) qRT-PCR and antibody tests after the summer holidays between August 25 and September 30, 2020. (3) Antigen tests after Christmas holidays between January 4 and February 5, 2021.

**Figure 1:**
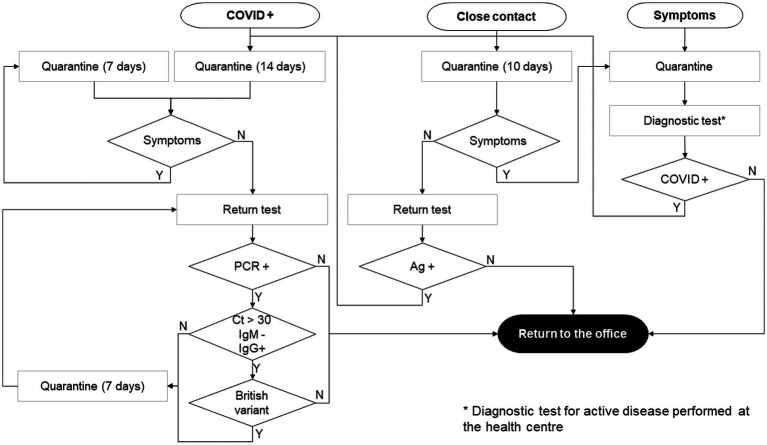
Return to the office flow chart. Depending on the initial state (COVID-positive, contact with positive, and presence of compatible symptoms), the return to the office strategy was adapted to minimize the risk of outbreaks. Ag, Antigen test; Ct, qRT-PCR cycles; IgM, IgM antibody serologic test; IgG, IgG antibody serologic test.

To minimize the probability of occupational infections, the minimal conditions needed to be approved to return to the office were more restricted in HQ than those defined by the Spanish Ministry of Health (10), as shown in the flow chart (Figure 1).

### Diagnostic tests

Different tests were used depending on technological development. (1) qRT/PCR: samples obtained by nasopharyngeal swabs for the study of the N1 and N2 sequences of the viral gene of the nucleocapsid 2019-nCoV, with the human gene RPP30 as a control (18). (2) Antigen tests: PANBIO™ COVID-19 Ag Rapid Test Device from Abbott, available since October 2020. Sample from nasopharyngeal swab (19). (3) IgM and IgG antibody rapid test: qualitative chromatographic immunoassay from blood sample. Hangzhou AllTest Biotech Co., Ltd. 2019-nCoV IgG/IgM rapid test cassette (20). (4) IgM and IgG quantitative serologies: ELISA with immunoassay reagents against proteins N of the SARS-CoV-2 virus and CLIA with immunoassay reagents against Receptor Binding Domain of the subunit S1 from the spike protein of the virus SARS-CoV-2. qRT-PCR and quantitative serologies were performed by EUROFINS/MEGALAB® laboratories. qRT/PCR, IgM and IgG rapid tests and antibody serologies were available since April 2020, while rapid antigen tests were available since October 2020.

### Patient and public involvement

Patients and the public were not involved in the design, conduct, reporting, or dissemination plans of our research. Workers provided informed consent to share the data gathered for epidemiological purposes, and the data were fully anonymized and filed as confidential.

## Results

### Evolution of the diagnostic tests during the pandemic

**Figure 2:**
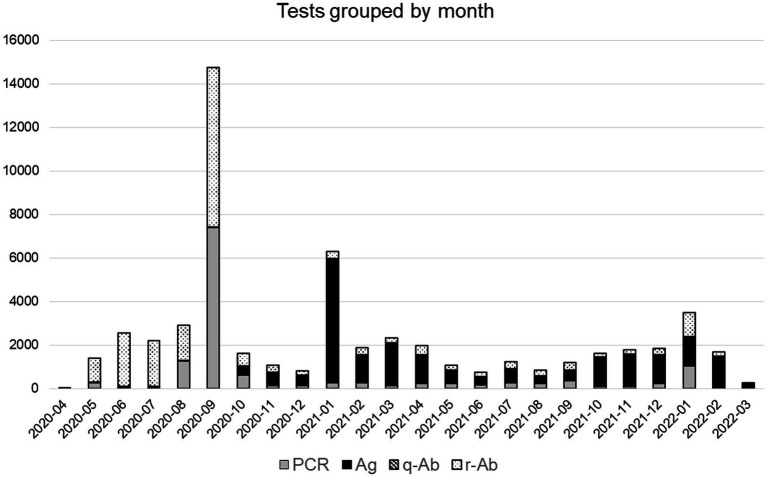
Monthly COVID-19 tests performed in headquarters depending on their type. qRT-PCR (PCR): gray, antigens (Ag): black, quantitative IgM/IgG antibodies serologies (q-Ab): dashed lines, and qualitative IgM/IgG antibodies rapid tests (r-Ab): dotted.

A total of 55,789 diagnostic tests were applied at the headquarters, with a total of 35,105 diagnostic tests of current infection (13,556 qRT-PCR and 21,546 antigen rapid tests) and 20,687 IgM and IgG antibody tests (20,305 rapid tests and 382 quantitative serologies). The monthly tests description is shown in Figure 2. Regarding the massive testing approach, during the post-confinement return, 5,184 tests were applied (318 qRT-PCR, 4,721 antibody rapid tests, and 145 quantitative serologies). During the 2020 summer holiday return, 15,752 tests were performed (7,910 qRT-PCR, two antigen rapid tests, 7,824 antibody rapid tests, and 16 quantitative serologies). During the 2020–21 winter holiday return, 5,783 tests were applied (24 qRT-PCR, 5753 antigen rapid tests, and six antibody rapid tests).

Regarding the tests performed exclusively at the Headquarters, 7,606 employees (67.2% of the total population) were tested using qRT-PCR, 8,883 (78.5%) with antigen rapid tests, 9,155 (80.9%) with IgM/IgG rapid tests, and 297 (2.6%) with quantitative IgM/IgG serologies. Details are presented in Table 1.

**Table 1:** Number of diagnostic tests performed at Headquarters and medical status of the total headcount (HC).

		Women	Men	Total	% over total HC	Chi^2^ *p* value
Headcount (HC)		4,561 (40.3%)	6,761 (59.7%)	11,322	-	
	COVID +	1,703 (42.3%)	2,323 (57.7%)	4,026	35.6%	< 0.001
	Employees with COVID medical records	2,708 (42.0%)	3,737 (58.0%)	6,445	56.9%	< 0.001
COVID infections (including reinfections)		1,762 (42.2%)	2,418 (57.8%)	4,180	-	
qRT-PCR		3,104 (40.8%)	4,502 (59.2%)	7,606	67.2%	
	qRT-PCR+	198 (37.6%)	329 (62.4%)	527	4.7%	0.1280
Antigens		3,616 (40.7%)	5,267 (59.3%)	8,883	78.5%	
	Antigen+	72 (41.4%)	102 (58.6%)	174	1.5%	0.9169
Antibody rapid tests		3,699 (40.4%)	5,456 (59.6%)	9,155	80.9%	
	IgG+	1,169 (41.5%)	1,649 (58.5%)	2,818	24.9%	0.1675
	IgM+	119 (38.8%)	188 (61.2%)	307	2.7%	0.5911
Quantitative serologies		129 (43.4%)	168 (56.6%)	297	2.6%	
	IgG+	42 (45.2%)	51 (54.8%)	93	0.8%	0.7801
	IgM+	11 (61.1%)	7 (38.9%)	18	0.2%	0.1883

Relative frequencies on each test or status are separated by sex, and relative frequencies over the total headcount. Test results does not include analyses taken externally.

### Evolution of COVID-positive cases and close contacts

The first positive case since April 2020, was recorded on July 13, 2020. A total of 158 positive cases were first diagnosed at headquarters, 21 were identified during the 2020 summer holiday return, 8 during the 2020–21 winter holiday return, and 129 through standard medical follow-ups. During the first wave, 66% of the personnel that had IgG-positive results declared being symptomatic (data not included in the tables, see *limitations* section), while 100% of the personnel with infections detected in the massive screenings already showed symptoms.

Concerning the updated COVID-related medical records, 24,899 updates and 19,003 histories were registered for 6,445 different employees. A total of 4,247 updates (17% of the total) corresponded with diagnosed COVID-positive cases, 3,550 updates (14%) corresponded with quarantines from contact with a confirmed COVID-positive case, 1,210 (5%) corresponded with informed compatible symptoms, 1,235 (5%) corresponded with other types of quarantines or isolation, such as risk-related factors, and the remainder of the cases (59%) corresponded with other types of updates.

Regarding quarantines, there were 2,771 histories of contact with a confirmed COVID-positive case, and 259 (9.4%) evolved to COVID-positive cases. A total of 806 histories of compatible symptoms of those in quarantine or isolated were registered, and 143 (17.7%) evolved into COVID-positive cases. In addition, 1.7% of the infections remained positive after 14 days of isolation, including both vaccinated and unvaccinated professionals.

**Figure 3:**
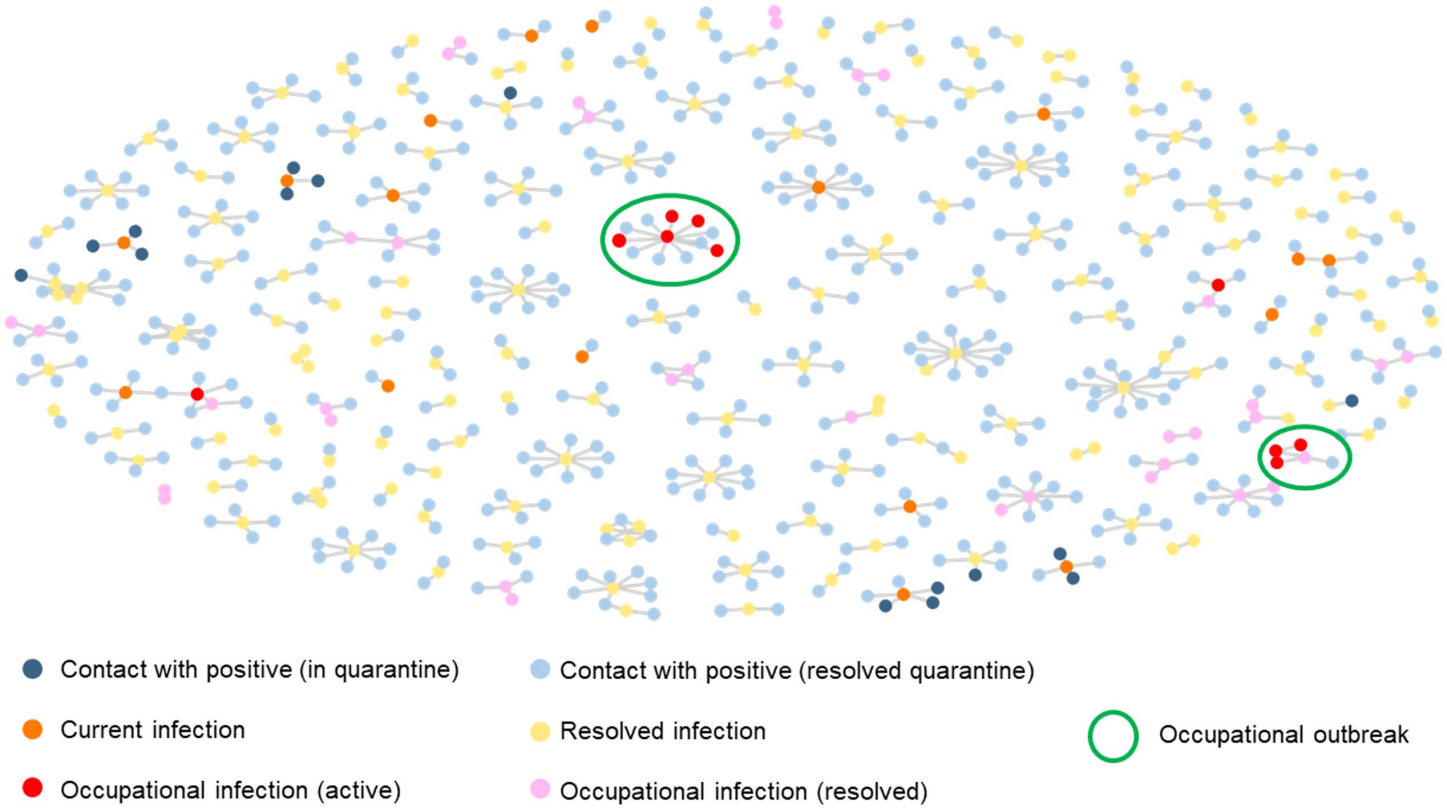
Networking diagram of confirmed cases of COVID-19 infections and occupational contacts. Current contacts and infections are shown in bright colors, resolved quarantines and infections are shown in dimmed colors, and occupational outbreaks (three or more related infections) are rounded. Each node represents an employee that had contact with an infected coworker and are connected through lines, being central nodes the first case of infection. Contacts who developed the disease are shown in orange, yellow, red, and pink. Infections exclusively attributed to occupational contacts are shown in red and pink.

One-thousand, eight-hundred and three contacts with COVID-positive professionals were identified by the tracking and medical team with the assistance of automatic algorithms. Ninety-one additional contacts between employees outside the workplace were manually included. Four-thousand, one-hundred and eighty COVID-19 infections were registered, of which 154 corresponded to reinfections, 4,105 (98.1%) cases were explained by external factors, and 75 (1.9%) were caused by occupational contacts (Figure 3).

### Comparison with the regional COVID incidence

**Figure 4:**
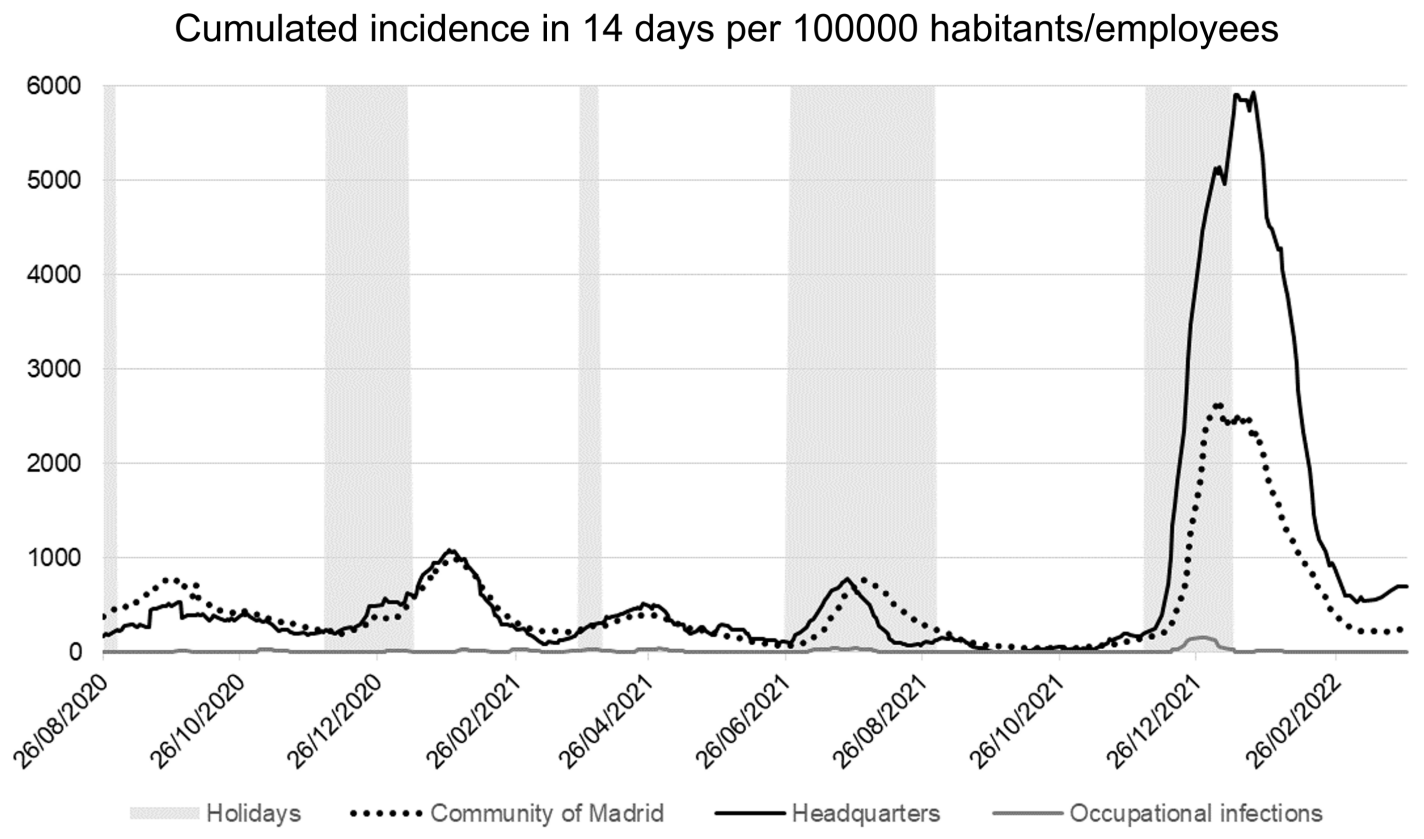
14-day cumulative incidence related with the population of the perimeter (habitants or employees). Community of Madrid incidence (dotted line), HQ (solid black line), and occupational infections (solid gray line). Holiday periods (summer, Christmas, and Holy week) are shaded, showing a relationship with outbreak waves. The first wave (between March and June 2020) is not represented due to the absence of validated data, as the diagnostic tests were only available for severe cases.

Figure 4 shows the 14-day cumulative incidence (CI) of COVID-19 infections during the last 14 days relative to 100,000 habitants for the Community of Madrid, or per 100,000 employees in the case of HQ (occupational and total infections). Six well-defined waves were identified, with a relationship between the peaks and summer, winter, and holy week holidays. During the first wave, diagnostic tests were only available for severe infections (21); therefore, they were not represented because of the lack of valid data.

A high positive Pearson correlation (*r* = 0.94) was found between the CI of COVID-positive cases in the last 14 days per 100,000 habitants in Madrid and the incidence per 100,000 employees in the HQ. However, a low correlation was found (*r* = 0.47) between CI in Madrid and COVID-positive cases due to occupational infections. Before the sixth wave, between December 1 and March 10, 2022, the correlation between the CI of the Region of Madrid and cases due to occupational infections was even lower (*r* = 0.35).

Additionally, Student’s T test and Fisher’s F tests analyses between the second and fifth waves (August 2020–December 2021) indicated that the means and variances of the CI in Madrid and HQ are equal (*t*-test value of *p* = 0.0634, confidence = 95%; F-test value of *p* = 0.9686, confidence = 95%). However, when analyzed including the sixth wave, the differences were significant in both tests (*t*-test value of *p* < 0.001, confidence = 95%; F-test value of *p* < 0.001, confidence = 95%), showing a peak of 2667.70 positives per 100,000 habitants in Madrid, in contrast with 5935.35 positives per 100,000 employees from headquarters (Figure 4).

Finally, only six occupational outbreaks were detected in the HQ between the second and sixth waves in Spain. The first and second outbreaks were caused by the British variant B1.1.7. and from third to sixth by the Omicron variant. The first outbreak consisted of one case and three occupational infections, the second and fourth with one positive case and four occupational infections, and the remainder of the outbreaks with one positive case and two occupational infections.

## Discussion

The aim of this study was to document the measures taken in place to control the spread of the SARS-CoV-2 virus in the workplace, independent of the transmissibility of the different variants, before and after vaccination, and the importance of the assistance of data science.

During this study, no academic reports about the incidence of the virus in sectors excluding healthcare or education were found, including this level of massive screenings and invested resources, indicating the importance of this work. Additional studies limit their field on recommendations toward working from home, wearing personal protective equipment, and performing diagnostic tests, but they do not include measures aided by data science in this sector (22–25).

There is no clear information regarding the incidence of COVID-19 among financial companies. However, some studies have explored the pandemic’s impact on small businesses.

During the first wave, there were no validated diagnostic tests reachable to the medical professionals of Santander, as qRT-PCR tests were reserved for severe cases (21, 26). The first valid tests available for non-hospital use since April 2020, were qRT-PCR, IgG/IgM antibody rapid tests, and antibody quantitative tests, with qRT-PCR being the gold standard to diagnose current infections.

Because of the high cost of qRT-PCR tests, when no sensitive antigen tests were available, IgG/IgM rapid tests were used to infer current infections, as suggested by some authors (27, 28). As indicated in this study, IgM rapid tests performed poorly in inferring current infections (16% concordance between IgM+ and qRT-PCR+ or Antigen+). However, prior to the availability of antigen tests, qRT-PCR was performed when employees tested positive for IgM. Once the Abbot® antigen tests were available, the screening strategy substituted the qRT-PCR with antigen tests. In case of doubt, qRT-PCR was also performed.

After the development of sensitive antigen tests, a clear tendency toward their use was observed in October 2020. The replacement of qRT-PCR with antigen tests resulted in substantial savings without compromising employee safety. It is worth highlighting that the cost of one qRT-PCR test was equivalent to 20 antigen tests, and the availability of the results were not dependent on laboratory equipment; therefore, a strategy for the use of antigen tests can be considered more cost efficient.

Scientific evidence in this work showed that the probability of infection was significantly lower in the workplace (1.9%), probably because of the diverse and simultaneous measures adopted in the offices, as well as the discipline toward the use of masks, hygiene, cleaning, and incremented air renovation. These measures could be relaxed in domestic environments, which might mean that working from premises may add an incremented protection factor against the probability of infection.

As previously mentioned, the high correlation of the incidences found between the Community of Madrid and the Headquarters’ population is not the same compared with the incidences of the region and occupational infections. In order to maintain a more conservative approach, the protocol used in this study was even more restrictive in terms of the definition of infection due to occupational contacts, as the medical staff preferred to have an oversized perspective (by considering the doubtful source of contact as occupational contacts) on the magnitude of the cases rather than to relax the measures. Nevertheless, in the databases and other levels of management, the doubtful cases were easily identified and labeled. Only in the very certain cases of infection from a private life context (conjugal, familiar, etc.), the infection was defined as non-occupational. This is the most plausible explanation for the increase of occupational infections during the sixth wave. At that time, the high rate of incidences observed in the region obstructed the analysis of the real origin of the contact. As previously mentioned, the staff decided to maintain a conservative approach and consider these contacts as internal; still, the occupational incidence was significantly lower.

Moreover, during the sixth wave (between December 2021 and February 2022), the highest rate of incidences registered for the population in HQ was 2.2 times the highest rate of incidences in the region. This significant difference can be explained by the fact that antigen self-test kits became available for the general population during that period, and the positive results were not reported to the health authorities and, therefore, were not included in the region’s statistics. However, positive results from self-tests were reported to the company’s medical staff. Consequently, it can be deduced that the incidence reported internally is a more accurate measure of the incidence in the region. This validated the impact of the sixth wave in addition to the relaxation of the measures taken by health authorities after 84% of the Spanish population was vaccinated (29).

Based on the data, the authors propose that a targeted action approach is more efficient than massive screening. Data show that there were more infections detected from medical-based algorithm screening (81.8%) than those found during the return from holidays’ massive tests (18.2%). Relative to the 4,180 infections registered, these represent only 0.69% of the total positives registered. It is also worth highlighting that all the infections detected during the massive screenings presented symptoms during the illness, which means that they would have been identified as well by the assisted protocol. However, during the first wave, only two-thirds of the confirmed COVID-positive cases by IgG rapid tests mentioned that presented symptoms in the previous months. Besides these numbers were not included in the current report due to the lack of quality, the authors propose that there might be a psychological bias during winter months and COVID-related symptoms might be ignored or forgotten, in contrast to presenting cold-like symptoms in summer.

Finally, the authors emphasize that a prevention-oriented strategy is highly efficient and sufficient to prevent occupational infections even in the context of undeveloped vaccines and treatments.

### Limitations of this study

This study has potential limitations due to the emergency state and the need to rapidly adapt to current protocols.

First: During the first wave of the pandemic, qRT-PCR tests were only available in hospitals to identify and treat severe COVID-19 cases (26). In consequence, the data gathered during this wave was not included into the statistics.
Second: The identification of the COVID-19 strain was not possible for all cases, therefore, the authors decided to consider a 21-day incubation window based on the initial recommendations of the Spanish Government and the Spanish Ministry of Health (10).
Third: At the beginning of the pandemic, a SIR model (susceptible, infected, and recovered) was initially tested; however, the results were inconclusive due to the lack of information to accurately calculate the variables and the presence of diverse strains with different reproduction rates, incubation periods and recovery rates. Therefore, the protocol was redefined toward a more descriptive, preventive and reactive strategy rather than predictive.
Fourth: During the sixth wave, the magnitude of the outbreak in the region made impossible to univocally identify the infections caused by occupational contacts, which means that any possible contact with an infected collaborator that ended in another infection was considered as occupational, even if the contagion occurred with the same probability from non-work factors. Consequently, the incidence rate due to occupational contacts might be oversized.

## Conclusion

In a context of health emergency and rapid adaptation to an unknown virus, a correct gathering of relevant data is mandatory.

If there are no vaccines or efficient treatments, the most effective strategy is to define several lines of defense: (1) To identify the life-threatening susceptible persons and send them to work from home until there is a vaccine or efficient treatment; (2) To isolate and send to work from home the suspected (symptomatic and closed contacts) and COVID-positive cases. (3) To include the COVID-positive cases into the database and calculate their possible contacts, as well, to manage a phone interview and confirm the list of the contacts. (4) To inform and restrict the access to potential and confirmed cases as soon as possible.

It is highly recommended to adopt an analytical decision-making process to avoid bias and deploy a technological infrastructure as fast as possible. To make it possible, it is highly recommended to follow a training course aimed for the medical and tracking teams with the following items: (1) Introduction to decision-making analytics and bias avoidance. (2) Data input training: mandatory fields and data quality check. (3) Dashboard uses and theorical fundamentals of Machine Learning algorithms. Additionally, and to increase personnel collaboration, it is recommended a webinar including basic concepts of hygiene measures and how their data is being used to ensure a safe workplace. The webinars aimed to the personnel resulted in higher engagement from the employees and was demonstrated in internal surveys.

A descriptive and reactive strategy can be a good solution if there is no enough information to make accurate predictions. Therefore, this methodology can also be applied in future pandemics of similar characteristics, especially with airborne transmission diseases.

In summary, the joint management of the pandemic between medical and technological professionals resulted in highly adapted and flexible protocols, which effectively blocked the transmission of the virus among employees working from premises and ensured the continuity of business as usual during the pandemic.

## Data availability statement

The data supporting the conclusions of this article will be made available by the authors, upon reasonable request. Due to local regulations, restrictions apply regarding the identification of the subjects and the data will be anonymized.

## Ethics statement

Written informed consent was obtained from the individual(s) for the publication of any potentially identifiable images or data included in this article.

## Author contributions

AP-H made the data collection, analysis, and interpretation, programmed the algorithms, contributed to the design of the work, and wrote the article. LR-B, LG-P, JM-G, MM-R, RD-G, and JM-S contributed with the design of the work, data collection, and revised the article. All authors contributed to the article and approved the submitted version.
